# Volumetry based biomarker speed of growth: Quantifying the change of total tumor volume in whole-body magnetic resonance imaging over time improves risk stratification of smoldering multiple myeloma patients

**DOI:** 10.18632/oncotarget.25402

**Published:** 2018-05-18

**Authors:** Markus Wennmann, Laurent Kintzelé, Marie Piraud, Bjoern H. Menze, Thomas Hielscher, Johannes Hofmanninger, Barbara Wagner, Hans-Ulrich Kauczor, Maximilian Merz, Jens Hillengass, Georg Langs, Marc-André Weber

**Affiliations:** ^1^ Diagnostic and Interventional Radiology, University Hospital Heidelberg, Heidelberg, Germany; ^2^ Department of Computer Science, Technical University of Munich, Munich, Germany; ^3^ Division of Biostatistics, German Cancer Research Center (DKFZ), Heidelberg, Germany; ^4^ Department of Biomedical Imaging and Image-Guided Therapy, Computational Imaging Research Laboratory, Medical University of Vienna, Vienna, Austria; ^5^ Department of Medicine V, Multiple Myeloma Section, University of Heidelberg, Heidelberg, Germany; ^6^ Department of Medicine, Roswell Park Comprehensive Cancer Center, Buffalo, NY, USA; ^7^ Institute of Diagnostic and Interventional Radiology, University Medical Center Rostock, Rostock, Germany

**Keywords:** volumetry, speed of growth, biomarker, risk stratification, smoldering multiple myeloma

## Abstract

The purpose of this study was to improve risk stratification of smoldering multiple myeloma patients, introducing new 3D-volumetry based imaging biomarkers derived from whole-body MRI.

Two-hundred twenty whole-body MRIs from 63 patients with smoldering multiple myeloma were retrospectively analyzed and all focal lesions >5mm were manually segmented for volume quantification. The imaging biomarkers total tumor volume, speed of growth (development of the total tumor volume over time), number of focal lesions, development of the number of focal lesions over time and the recent imaging biomarker ‘>1 focal lesion’ of the International Myeloma Working Group were compared, taking 2-year progression rate, sensitivity and false positive rate into account.

Speed of growth, using a cutoff of 114mm^3^/month, was able to isolate a high-risk group with a 2-year progression rate of 82.5%. Additionally, it showed by far the highest sensitivity in this study and in comparison to other biomarkers in the literature, detecting 63.2% of patients who progress within 2 years. Furthermore, its false positive rate (8.7%) was much lower compared to the recent imaging biomarker ‘>1 focal lesion’ of the International Myeloma Working Group.

Therefore, speed of growth is the preferable imaging biomarker for risk stratification of smoldering multiple myeloma patients.

## INTRODUCTION

Multiple Myeloma (MM) precursor diseases are defined by the detection of M-protein or the presence of clonal plasma cells within the bone marrow. To account for the different extent of those findings and their unequal risks of progression to active MM, these precursor states are grouped into monoclonal gammopathy of undetermined significance (MGUS) and smoldering multiple myeloma (SMM) [1]. However, even just within the group of SMM patients, the prognosis has been demonstrated to be extremely heterogeneous: While approximately 20% of SMM patients develop active MM within the first 2 years, 25% have not progressed after 10 years, indicating that further risk stratification and different management for subgroups within SMM are needed [2].

The International Myeloma Working Group (IMWG) updated the definition of MM in 2014 with the purpose of including a subset of high-risk SMM patients, who show a high risk for imminent development of end organ damage defined by the CRAB-criteria, into the group of MM patients requiring therapy. To do so, they searched for so called biomarkers of malignancy, which isolate a subset of patients who show an 80% probability of progression to MM (defined by CRAB criteria only) within the next 2 years [3].

A large prospective study was able to show that high-risk SMM patients do benefit from early therapy, with lenalidomid and dexamethasone significantly extending the time to development of CRAB-criteria [4]. Even though high-risk SMM patients were not defined by imaging in this study, this emphasizes the value of optimal risk stratification for SMM patients. However, the current MRI biomarker from the IMWG, i.e. the presence of more than 1 focal lesion (FL) of greater than 5mm in size, has repeatedly failed to reach an 80% 2-year progression rate (2yrPR) [5, 6]. Also, to our best knowledge, the sensitivity and false positive rate (FPR) of this criterion has never been assessed, lacking evidence about which proportion of patients that will progress within 2 years is actually detected by the biomarker ‘>1 focal lesion’ (>1FL).

Therefore, the purpose of this study was to establish and assess new, volumetry based biomarkers derived from whole-body MRI (wb-MRI) and to define the best possible MRI biomarker for risk-stratification of SMM patients, taking the 2yrPR as well as sensitivity and FPR into account.

## RESULTS

### Analysis of the predictive value of longitudinally assessed total tumor volume, speed of growth, number of focal lesions and the development of the number of focal lesions over time for the risk of progression

We observed a significant correlation between the total tumor volume (TTV) and the time to progression (TTP). Quantitative analysis showed that an increase of the TTV by the factor of 10 leads to an increase of the risk of progression by 65%. Using a cutoff of 7220mm^3^ TTV for risk stratification into a high-risk and a low-risk group exactly fulfilled the demand of the IMWG to isolate a high-risk group with a 2yrPR of 80%.

**Figure 1:**
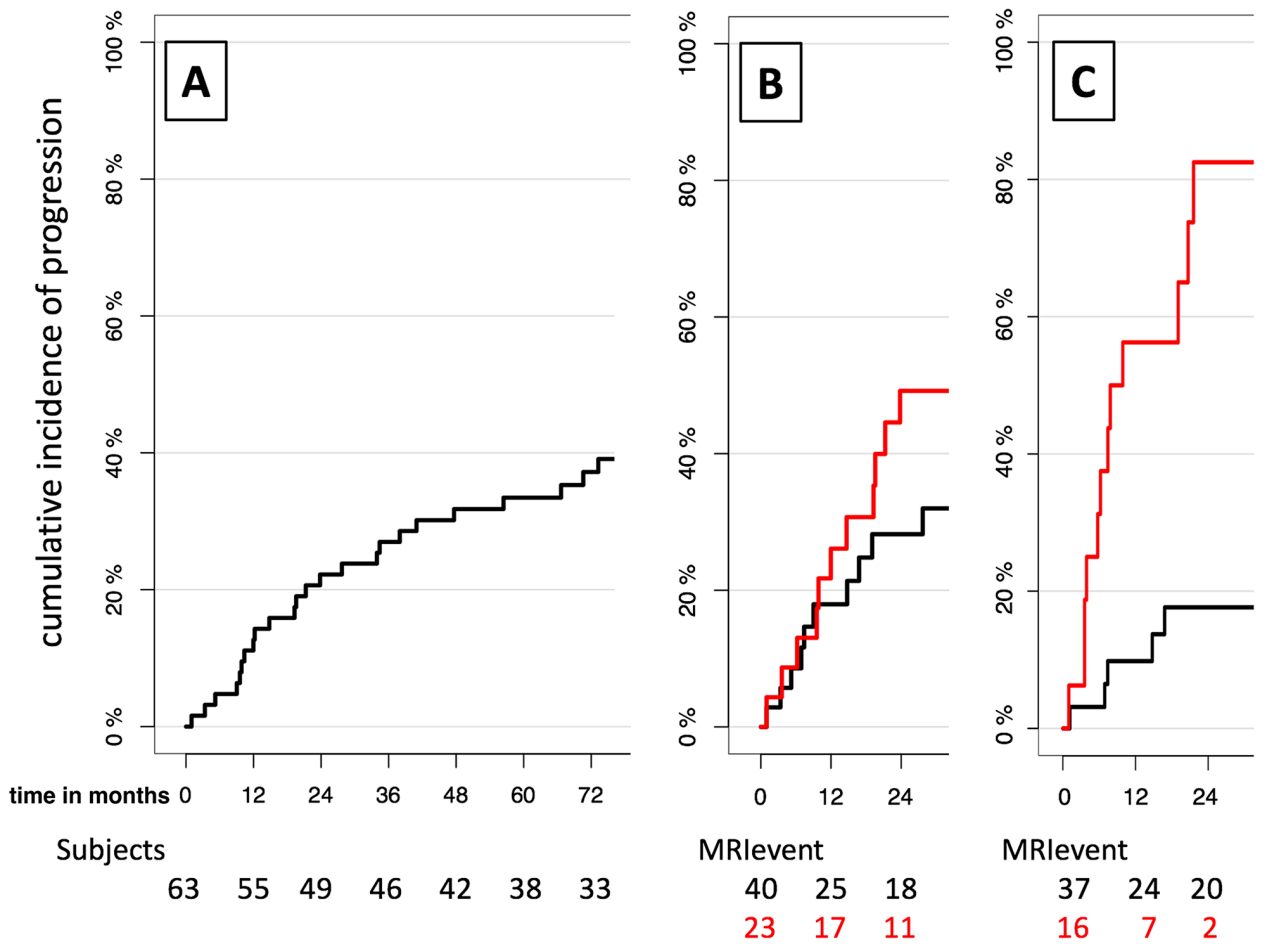
Development of risk stratification for SMM patients by imaging biomarkers: without imaging biomarker **(A)**, with current imaging biomarker >1FL **(B)** and with new imaging biomarker SOG **(C)**. A: Cumulative incidences of progression for all SMM patients from initial MRI when no risk stratification by an imaging biomarker is performed. Time is given in months from initial MRI. B-C: Cumulative incidences of progression for high-risk group (red) and low-risk group (black). Risk stratification into high- and low-risk group is performed by the current IMWG imaging biomarker >1FL in B, and the new volumetry-based imaging biomarker >114mm3/month SOG in C. Time is given in months from the MRI with biomarker-event (MRI-event), defined as the first time the biomarker-cutoff is overstepped, or from last follow-up MRI when no biomarker event occurred during the follow-up. Below, the number of patients in each group “at risk” is given. The SOG reaches both higher progression rates in the high-risk group and lower progression rates in the low-risk group compared to >1FL.

Performing quantitative analysis for speed of growth (SOG), we observed a 37% increase of the risk of progression for each additional 100mm^3^/month SOG, which was also a significant correlation. The cutoff to fulfill the IMWG demand was located at 114mm^3^/month: Patients that once showed a SOG of more than 114mm^3^/month had a 2yrPR of 82.5% from this point in time (Figure 1). By adjusting the SOG cutoff, achieving 2yrPR over 90% in the high-risk group was possible.

According to our data, adjusting the cutoffs for the non-volumetry based MRI-biomarkers number of focal lesions (nFL) and the development of the number of focal lesions over time (dev-nFL) also allowed for isolation of a subset with at least 80% 2yrPR. Performing risk stratification based on the number of FLs, a cutoff of ≥5 FL isolated a high-risk group with a 2yrPR of 87.5%. For the development of the number of focal lesions, patients who showed ≥2 new FL per year had a 2yrPR of 85.2%.

Table 1 shows the different biomarkers assessed in this study, the current MRI biomarker (>1FL; Figure 1B), their cutoffs and summarizes their performance according to the IMWG criterion and both sensitivity and FPR. SOG showed the highest ability to discriminate patients that will progress within the next two years with a sensitivity of 63.2%, exceeding the current imaging biomarker >1Fl by 14.9%. In addition, the FPR of the SOG was less than a third of the biomarker >1FL. FPRs of nFL and dev-nFL were even lower than the one of SOG, however their ability to detect patients that will progress within the next 2 years was very low compared to the SOG.

**Table 1:** Overview of MRI biomarkers given with cutoff, p-value for cutoff, 2yrPR of the high-risk group and performance at sensitivity and FPR

MRI-based biomarker	>1 focal lesion	Number of focal lesions	Development of number of focal lesions	Total tumor volume	Speed of growth
Cutoff	>1	≥5	≥2	≥7220	≥114
Unit	FL	FL	new FL/year	mm^3^	mm^3^/month
p-value	<0.001	<0.001	<0.001	<0.001	<0.001
2-year progression rate	49.2%	87.5%	85.2%	80.0%	82.5%
Sensitivity	48.3%	26.5%	36.2%	47.0%	63.2%
False positive rate	29.5%	2.7%	4.2%	8.0%	8.7%
n patients assessed	63	63	53	63	53
n showing biomarker	23	8	9	15	16

Additionally, the number of patients that could be assessed with the biomarker and the number of patients presenting the biomarker within the follow-up is given.

To compare the ability of prognostic discrimination between the MRI biomarkers without cutoffs, Harrell's c-index was calculated for each biomarker, based on all longitudinally assessed biomarker values (Table 2). SOG showed the highest c-Index, stating that it has the best ability of prognostic discrimination among all tested MRI biomarkers.

**Table 2:** Harrell's c-Index for the MRI-biomarkers nFL, dev-nFL, TTV and SOG

MRI-based biomarker	Harrell's c-Index
Number of focal lesions	0.788
Development of the number of focal lesions	0.639
Total tumor volume	0.791
Speed of growth	0.825

Supplementary Figure 1 visualizes the complete follow-up of all patients showing focal lesions, displaying all wb-MRIs in a timeline and stating which biomarker was assessed as positive at the corresponding MRI.

### Multivariate analysis

Multivariate models were calculated in order to investigate whether TTV and SOG have independent prognostic value. Initial M-protein (M-protein t1) ≥20g/l, which had a significant effect on progression in our cohort, as well as nFL and development of new lesions vs. no development of new lesions were included in the multivariate analysis.

In a basic multivariate model for initial TTV (TTVt1) and initial M-protein, both were significant risk factors (Table 3A).

**Table 3:** Multivariate analysis

	Variable	HR	95% CI	p-value
A	***M-protein t1 ≥20 g/l***	***2.77***	***1.11, 6.89***	***0.02869***
	***TTVt1***	***1.44***	***1.00, 2.08***	***0.04793***
B	**M-protein t1 ≥20 g/l**	1.32	0.50, 3.51	0.57340
	***SOGevent (cutoff 0)***	***8.42***	***2.49, 28.45***	***< 0.001***
	logTTVt1	1.14	0.82, 1.58	0.43855
C	**M-protein t1 ≥20 g/l**	1.34	0.51, 3.55	0.55415
	***SOGevent (cutoff 0)***	***8.18***	***2.39, 28.02***	***< 0.001***
	nFL t1 >0	1.60	0.53, 4.90	0.40675
D	**M-protein t1 ≥20 g/l**	0.96	0.32, 2.84	0.93930
	***SOGevent (cutoff 114)***	***42.06***	***8.14, 217.33***	***< 0.001***
	logTTVt1	0.81	0.52, 1.27	0.35731
E	**M-protein t1 ≥20 g/l**	0.92	0.31, 2.73	0.88372
	***SOGevent (cutoff 114)***	***30.22***	***6.09, 149.80***	***< 0.001***
	nFL t1 >0	0.72	0.15, 3.46	0.68534
F	***TTV***	***1.57***	***1.18, 2.09***	***0.0018***
	***nFL***	***1.22***	***1.12, 1.32***	***< 0.001***

Significant findings are given in bold and italic letters.

Including initial M-protein ≥20g/l, initial tumor load and overstepping the cutoff of SOG>0 (first time overstepping certain SOG cutoff is called SOGevent in the following), this SOGevent always remained an independent, significant risk factor, while initial M-protein and initial tumor load lost significance. This was independent of whether initial tumor load was included continuously (logTTVt1) or categorially (nFL t1 >0 vs. nFL t1 =0) (Table 3B-3C). Applying the SOG>114mm^3^/month cutoff that we introduced to fulfill the biomarker criteria instead of SOG>0 did not change the significance of the parameters in this analysis: SOGevent with cutoff 114mm^3^/month remained an independent, significant risk factor (Table 3D-3E).

**Figure 2:**
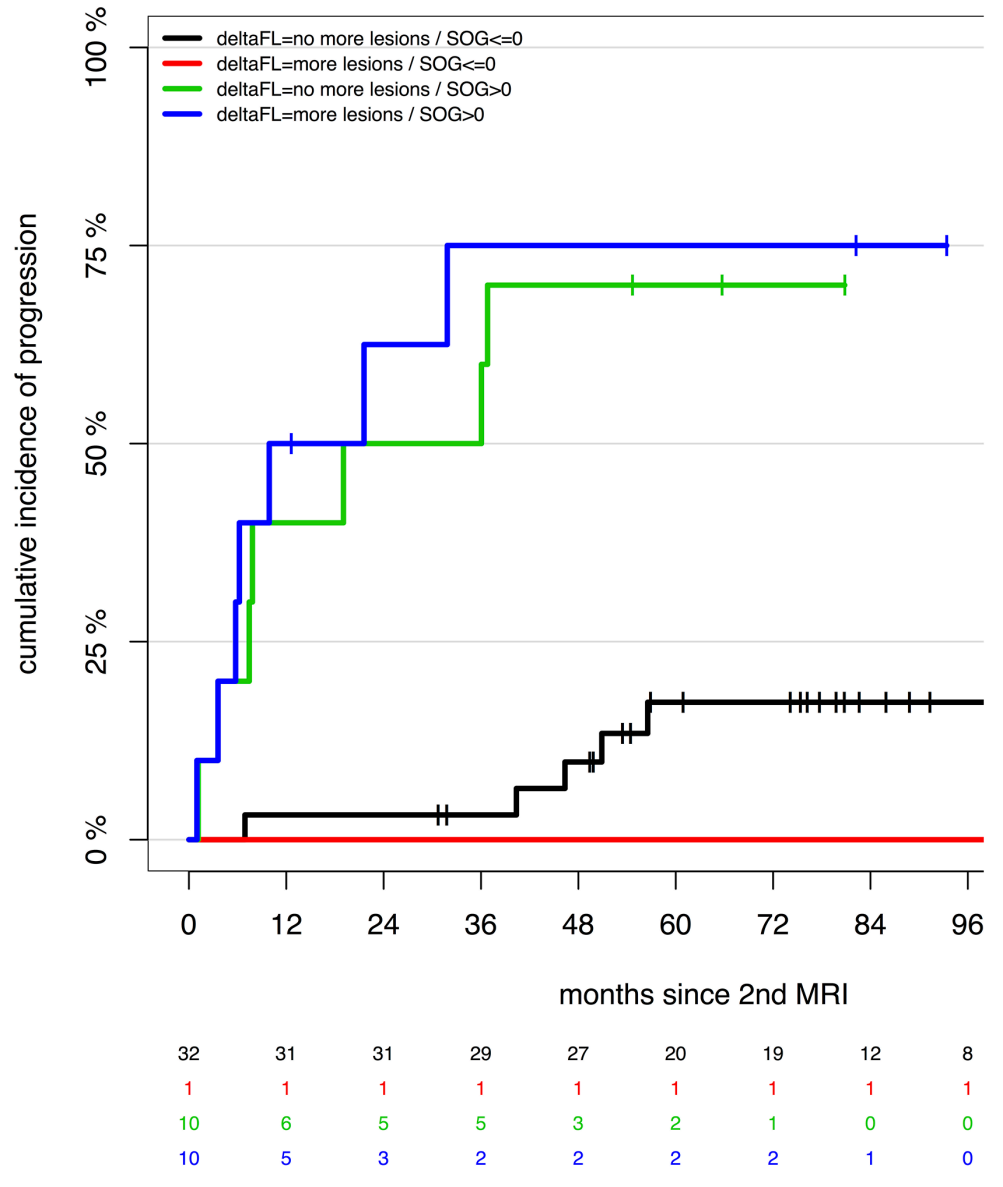
Speed of growth and appearance of new focal lesions Groups are simultaneously stratified for SOG at 2^nd^ MRI >0 vs. SOG at 2^nd^ MRI ≤0 and new focal lesions appearing at 2^nd^ MRI vs. no new focal lesions appearing at 2^nd^ MRI (deltaFL>0 vs. deltaFL=0, respectively). Groups with SOG>0 show a higher risk of progression than groups with SOG≤0, no matter whether the SOG>0 is caused by appearance of new focal lesions (deltaFL>0, blue, p<0.0001) or just by growth of preexisting lesions (deltaFL=0, green, p<0.0001). In the subgroup with SOG>0, patients with new FL show a 28% higher risk of progression, which is not statistically significant in our cohort (p=0.65).

Another interesting question is whether the statistical significance of the SOG depends on whether it is caused by the appearance of new FLs or only by the growth of already existing lesions. To investigate this, we stratified groups by new lesions vs. no new lesions at the 2^nd^ wb-MRI and SOG>0 vs. SOG≤0 (Figure 2). Patients with SOG>0 showed significantly higher incidence of progression than patients with stable disease on wb-MRI (no new lesions and existing lesions not growing), independent of whether new lesions appeared or only existing lesions grew (each with p<0.0001). In the subgroup of patients with SOG>0, patients who developed new lesions had 1.28 times risk of progression compared to patients without new lesions, however this was not statistically significant (p=0.65).

When multivariate analysis was performed for TTV and nFL over all follow-ups, both remained as significant independent risk factors (Table 3F), also showing that the measurement of tumor volumes had additional prognostic value compared to the lesion count.

### Correlation of clinical data with TTV/SOG

We found no significant correlation between any lab parameter and the initial TTV (Supplementary Table 1). For the SOG at 2^nd^ MRI, a significant correlation was observed to the initial beta-2-microglobulin (B2MG) serum level (Table 4A) as well as to deltaHb between initial and 2^nd^ MRI (Table 4B). Results for all other parameters did not show significant correlations (Table 4A-4B).

Table 4Correlation between SOG and baseline disease parameters and their development

**Table:** 

4A		Parameter			n	rho		p-value	
		M-protein			28	0.15		0.4471	
		Plasmacell count			37	0.13		0.4426	
		Lactate dehydrogenase			45	0.15		0.3316	
		Creatinin			47	0.21		0.1601	
		Calcium			45	0.17		0.2671	
		Platelets			45	-0.05		0.7611	
		Leukocytes			45	0.17		0.2527	
		***B2MG***			***35***	***0.35***		***0.0423***	
		Hemoglobin			47	0.08		0.5738	
		Albumin			43	-0.1		0.5054	
		C-reactive protein			33	0.14		0.4465

**Table:** 

4B	Parameter		n	rho			p-value	
	delta M-protein		22	0.41			0.0548	
	delta Creatinin		43	-0.07			0.6698	
	delta Calcium		41	-0.12			0.4585	
	delta Platelets		21	-0.14			0.5404	
	delta Leukocytes		21	-0.27			0.2391	
	***delta Hemoglobin***		***43***	***-0.41***			***0.0067***	
	delta Albumin		34	-0.13			0.4473	

Table 4A shows correlation between baseline disease parameters and SOG at 2^nd^ MRI.

Table 4B states correlations between development of disease parameters between first and 2^nd^ MRI and SOG at 2^nd^ MRI. Significant findings are given in bold and italic letters.

## DISCUSSION

MRI has proven to be a suitable tool for risk stratification of SMM patients, using abnormal bone marrow pattern [7, 8], count of FLs [5, 6, 9, 10], development of MRI findings [6] or even parameters from dynamic contrast enhancement MRI (DCE-MRI) as criteria to isolate high-risk groups [11]. Additionally, it was shown that volumetry is the superior method of quantifying tumor burden [12, 13] and that volumetry can contribute to proper risk stratification in lung cancer screening [14, 15]. With these developments in mind, the present study introduces volumetry-based MRI biomarkers for risk stratification of SMM patients and demonstrates their superiority in comparison to other biomarkers, including the recent biomarkers of the 2014 IMWG guidelines.

### Performance of biomarkers tested in this study

While the biomarker >1FL failed to reach 80% 2yrPR, both non-volumetry-based (nFL, dev-nFL) and both volumetry-based (TTV, SOG) biomarkers were able to fulfill the IMWG criterion to isolate a high-risk group with ≥80% 2yrPR when cutpoints were adjusted. Taking sensitivity and FPR into account shows that >1FL performs strongly in detecting almost half of the patients that will progress within 2 years, but falsely assigns almost a third of patients who will not progress within 2 years to the high-risk group. This is by far the highest FPR and it results in possible harm to a high number of patients caused by side effects from unnecessary or too early therapy. With 63.2%, SOG shows by far the highest rate of detecting patients with progression within 2 years of all MRI biomarkers, while only assigning 8.7% of patients who are not progressing within 2 years falsely to the high-risk group. The TTV performs similar to SOG regarding FPR, but performs markedly worse regarding sensitivity. The non-volumetry-based biomarkers show the lowest FPRs but poor sensitivity, achieving only about half of the sensitivity of the SOG. Besides the performance on 2yrPR, sensitivity and FPR, Harrell's c-index additionally expresses the superiority of the SOG for risk stratification.

As a biological background for the superior performance of the volumetry-based biomarkers, it has recently been demonstrated that the maximum diameter of a focal lesion correlates with the proportion of site-specific mutations, indicating an association between the size of FLs and advance of tumor biology [16]. In contrast to nFL and dev-nFL, the volumetry-based biomarkers take the size of focal lesions into account and therefore include information about the current advance of tumor biology, which is lost when only the number of FLs is assessed.

### Performance of SOG in comparison with other biomarkers from the literature

The biomarker >1FL has been associated with a higher risk of progression [5, 6, 9, 10], but failed to isolate a high-risk group with ≥80% 2yrPR [5, 6], which is supported by our data.

With the criterion ‘radiological Progressive Disease’ (rPD) Merz et al. proposed an imaging biomarker evaluating development of both focal and diffuse pattern over time, resulting in a 2yrPR at the edge of 70% [6]. While the SOG performs better on the 2yrPR, it has to be remarked that we did not take the diffuse infiltration into account, which was of prognostic significance in some studies [7, 10]. A further improvement of the imaging biomarker might be achieved by combining SOG with parameters evaluating state and/or development of diffuse infiltration into a combined score.

Zamagni et al. performed risk stratification based on 18F-FDG PET/CT, where PET-positive findings defined by showing focal lesions or diffuse bone marrow involvement were associated with a 58% 2yrPR [17]. From the data shown in this publication, we calculated that the sensitivity of this biomarker was only 25%. In comparison to PET/CT, SOG not only performs much better on 2yrPR and sensitivity, but also does not have any radiation exposure.

To assess the absolute amount of contribution of the SOG to the detection of patients that will develop CRAB-criteria within two years by all biomarkers, the sensitivity of the other recent IMWG biomarkers has to be taken into account. For the serum free light chain ratio using a cutoff ≥100 the sensitivity for detecting patients with a progression within the first 2 years was 31.9% [18]. For the percentage of plasma cells in the bone marrow, Rajkumar reported that 3.2% of SMM patients showed a bone marrow plasma cell percentage ≥60% and that 95% of those progressed to MM within the first 2 years [19]. Thus, based on the estimation that 20% of SMM patients progress in the first 2 years [2], this results in a sensitivity of approximately 15%. Compared to 31.9% and approximately 15% estimated sensitivity of the other two current IMWG biomarkers, the SOG with 63% sensitivity contributes enormously to the overall detection of SMM patients that will progress within 2 years. Additionally, it is a non-invasive procedure which can easily be repeated annually or semiannually.

### Multivariate analysis

Using multivariate analysis, we demonstrate that overstepping a certain SOG cutoff (0 or 114mm^3^/month) is an independent, significant risk factor for progression to MM. The SOG as a parameter reflecting the dynamic of the disease has more prognostic impact than parameters based on only one point in time (such as TTV or nFL). This finding contributes to a collection of results in the body of literature that show a prognostic significance of longitudinal parameters both in imaging [6] and laboratory investigation [20–22].

The significance of SOG does not depend on whether it is exclusively caused by the growth of existing lesions or whether it also results from the arising of new lesions. A different pathomechanism might be the reason why in some cases only a few lesions exist and grow in a rather stationary manner, while in other patients many new lesions arise. With this in mind, it is an interesting finding that patients with new arising lesions at the 2^nd^ MRI have a tendency towards a more aggressive course of disease, even though this was not significant.

### Limitations

The small number of patients is a limitation of this study. However, it must be taken into account that only few centers performed MR imaging on SMM patients before the IMWG updated the disease definition of MM in 2014 [3]. Therefore, this is one of the largest cohorts with longitudinal MR-Imaging in untreated SMM patients. The retrospective design of this study has to be named as another disadvantage. Wb-MRIs were mainly scheduled by the treating physicians and did not follow a distinct schema such as follow up every 6 or 12 months, at least until 2014 when annual wb-MRIs became included in the routine assessment of SMM patients for the first 5 years from diagnosis. For these reasons, our results should be verified by a prospective study with a fixed follow-up schema and a larger number of patients. As the SOG is a longitudinal biomarker representing the dynamic of the disease like rPD, evolving M-protein or evolving Hb, it cannot be calculated at the initial examination, but start e.g. with the first follow-up after six months. Manual 3D-segmentation is currently a more time-consuming task than solely counting focal lesions. However, there is a development towards automatic tools for combined lesion detection and segmentation [23], which would reduce the workload for the determination of the SOG.

## MATERIALS AND METHODS

### Patients

In this study, 63 SMM patients (according to the 2003 guidelines [1]) who received at least one wb-MRI were analyzed retrospectively. All patients included in this study received initial wb-MRI between 2004 and 2011 at our center and received quarterly or at least semiannual clinical evaluations until they progressed to MM (n=23), received local therapy of FLs (n=4), systemic therapy due to the call of the treating physician without diagnosis of MM (n=6), died (n=2), or for at least 3 years from initial MRI in case none of the abovementioned events occurred (n=28). No patient had received a systemic therapy before or during the observation period; however 10 patients were included who had received local radiation therapy for a concomitant solitary plasma cell tumor before the first MRI. Volumetry was not performed on those irradiated solitary plasma cell tumors, but volumetry was performed on all focal lesions outside the radiation field. Progression was defined by development of myeloma defining events without biomarkers of malignancy, shortly the CRAB-Criteria [3]. Patients who received local therapy (radiation therapy or resection) of FLs or systemic therapy due to the call of the treating physician without diagnosis of MM were excluded from follow up from this time point, because the natural development of the TTV could have been affected by the therapy. Patients had a median age of 55 years (range 29-76 years) at initial MRI; 62% were male. Fifty-three patients received at least two (on average four) wb-MRIs, before progress occurred or they left observation. The median interval between MRI examinations was 13 months and the median observation time from first to last MRI was 46 months. Patients with one wb-MRI could not be included in analysis of the dynamic biomarkers speed of growth and development of the number of focal lesions over time. Approval had been received from the institutional ethics committee for retrospective analysis of imaging data from patients with monoclonal plasma cell disorders with waiver of informed consent. Parts of this cohort had been included in previous studies [6, 10, 24–26].

### Imaging protocols

All wb-MRI exams originating from two identical 1.5 Tesla MRI systems (Magnetom Avanto, Siemens Healthineers, Erlangen/Germany) at our site were included. The imaging protocol has been published before [24, 26] and comprised phased-array, body-matrix surface coils (Siemens Healthineers, Erlangen/ Germany). The sequence protocol included the following: T1-weighted turbo-spin echo sequences (repetition time (TR), 627 milliseconds [ms]; echo time (TE), 11 ms; section thickness (ST), 5 mm; acquisition time (TA), 2:45 min) and T2-weighted short-tau inversion recovery sequences (TR, 5300 ms; TE, 74 ms; ST, 5 mm; TA, 3:00) of the head, thorax, abdomen, pelvis, and legs in coronal orientation; T1-weighted turbo-spin echo sequences (TR, 621 ms; TE, 11 ms; ST, 3 mm; TA, 1:38 ) and T2*-weighted turbo-spin echo sequences (TR, 4000 ms; TE, 93 ms; ST, 4 mm; TA, 2:30) of the spine in sagittal orientation. The patients were positioned with their arms along the body. The examinations covered the region between the skull vertex and the mid-calves. Depending on the body height of the patient, the distal calves and the feet were not always included. The total image acquisition time was approximately 40 minutes. Contrast medium was not given.

### Volumetry

**Figure 3:**
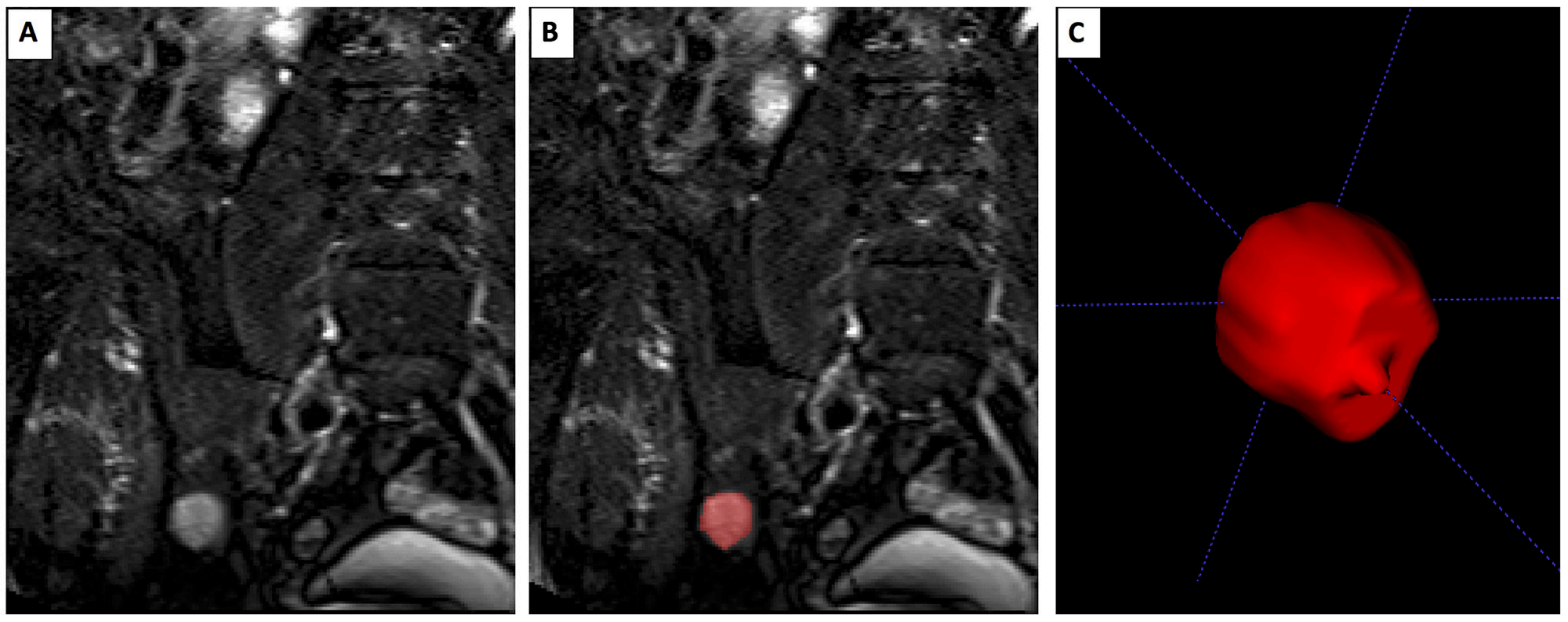
Segmentation of a focal lesion Extract of a T2-weighted coronal MRI sequence of abdomen/pelvis. **(A)** Native imaging with hyperintense focal lesion of the iliac bone in ITKSnap, **(B)** Image with focal lesion highlighted in red after 3D-segmentation was performed. **(C)** 3D-view of segmented focal lesion in ITK-SNAP.

ITK SNAP (Version 3.4.0, www.itksnap.org), a common software for the segmentation of anatomical structures in 3D medical images [27], was used for volumetry. Segmentation of all detected lesions >5mm was performed manually (Figure 3) by one research assistant who was supervised by a radiology resident experienced in musculoskeletal imaging. When performing volumetry, the examiners were blinded to clinical and prognostic information. In some cases, lesions were not delimitable anymore due to intensified diffuse bone marrow infiltration over time. We assume the tumor cells of those focal lesions did not disappear but are not measurable anymore. Since calculating no volume for those lesions (while the tumor load presumably is still present) would be incorrect, we performed a calculative correction for those unmeasurable lesions, using the volume of the lesion in the previous imaging as a replacement. FLs were measured in T1- and T2-weighted images if possible. As there was no systematic difference between T1 and T2 volumes, the average volume was used for further calculation. Adding up the volumes of all FLs results in the TTV. The SOG was defined by the difference of TTVs divided by the time between two consecutive MRIs.

### Clinical and laboratory examinations

Clinical and serological examination of included patients was performed every 3-6 months for the complete follow-up period in our outpatient clinic. Progression was defined by development of myeloma defining events without biomarkers of malignancy, shortly the CRAB-Criteria [3].

### Statistical analysis

Primary clinical endpoint was TTP, defined as time from 1st/2nd MRI to progression. Deaths without prior disease progression were considered as competing event. Cumulative incidence of progressive disease was estimated using Aalen-Johansen estimator accounting for death. Cause-specific Cox regression was used to assess the impact of imaging parameters on risk of progression. TTV was log10-transformed for prognostic analysis. Harrell's c-index was used to compare prognostic discrimination of different MRI biomarkers. Imaging parameters assessed during follow-up were analyzed as time-dependent variables in Cox regression. For multivariable Cox proportional hazard analysis, multiple imputation of missing M-Protein values was performed using MICE algorithm.

Imaging biomarker events were defined as the first time a certain threshold was exceeded. The 2yrPR in patients with imaging event was defined as the estimated proportion of patients with progression within 2 years after onset of imaging event. Cutoffs of imaging parameters were selected based on a 2yrPR of at least 80%. P-values of these cutoffs were adjusted for multiple testing of all possible cutoffs using Holm correction in order to control the family-wise error rate. For patients without imaging event during follow-up, time to progression was calculated from last MRI in cutoff analysis.

To assess the predictive discrimination of biomarkers for risk stratification beyond the 2yrPR after biomarker event, we assessed time-dependent sensitivity and FPR for disease progression after 2 years accounting for censored data [28].

Sensitivity here indicates the proportion of patients correctly identified to be at high risk, whereas the false positive rate, or 1 – specificity, indicates the proportion of patients incorrectly identified as being at high risk.

Spearman correlation coefficient was used to assess correlation between imaging and lab parameters. Analysis was performed with software R 3.4.

## CONCLUSIONS

Of all biomarkers derived from wb-MRI, SOG shows the best ability of prognostic discrimination and is the preferable imaging biomarker for risk stratification of smoldering multiple myeloma patients. Using a cutoff of 114mm^3^/month, it fulfills the IMWG demand of 80% 2yrPR of the high-risk group and detects by far the most patients that are fulfilling the CRAB-criteria within the following two years. Additionally, it shows a low FPR. Further studies on larger cohorts should be performed in order to confirm that the SOG is the best imaging biomarker and that SOG should be used for risk stratification of SMM patients or as a criterion for the definition of symptomatic disease with indication for systemic treatment.

## SUPPLEMENTARY MATERIALS FIGURES AND TABLES



## Abbreviations

B2MG: beta-2-microglobulin
CI: confidence interval
dev-nFL: development of the number of focal lesions over time
FPR: false positive rate
HR: hazard ratio
n: number
nFL: number of focal lesions
rPD: radiological progressive disease
SOG: speed of growth
ST: section thickness
t1: initial
TA: acquisition time
TE: echo time
TR: repetition time
TTP: time to progression
TTV: total tumor volume
