# Supplementary material for: Volumetry based biomarker speed of growth: Quantifying the change of total tumor volume in whole-body magnetic resonance imaging over time improves risk stratification of smoldering multiple myeloma patients
Source: Oncotarget. 2018 May 18;9(38):25254–64. doi: 10.18632/oncotarget.25402 (PMC5982766; doi:10.18632/oncotarget.25402)
Supplement: Supplementary file 1 [file oncotarget-09-25254-s001.pdf]

Volumetry based biomarker speed of growth: Quantifying the change of total tumor volume in whole-body magnetic resonance imaging over time improves risk stratification of smoldering multiple myeloma patients

SUPPLEMENTARY MATERIALS

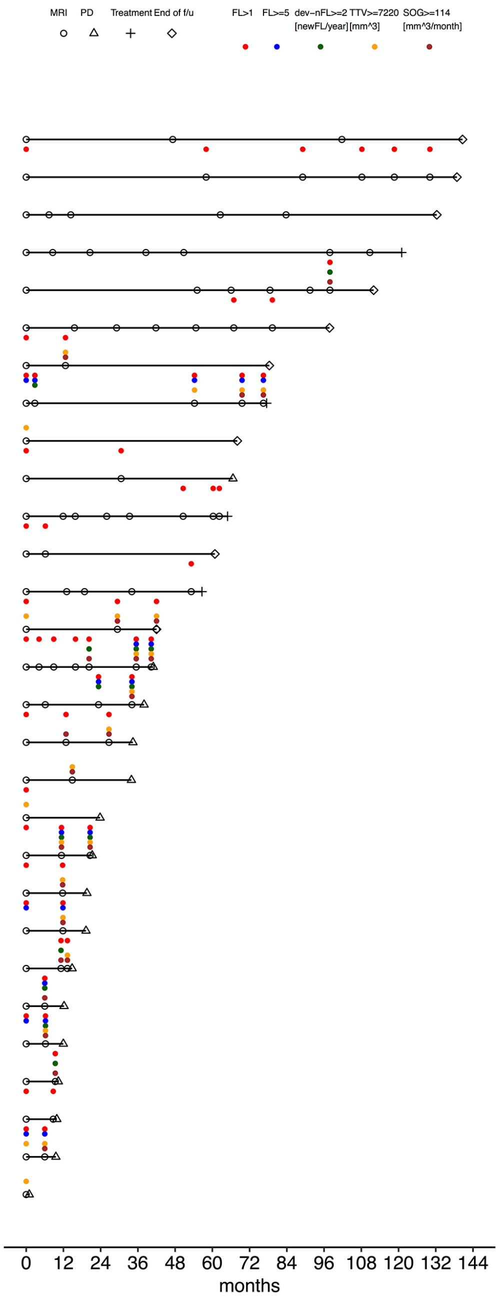

mean SOG [mm^3/ month]	max SOG [mm^3/ month]	max TTV [mm^3]	nFL first	nFL last	mean dev-nFL [newFL/ year]	max dev-nFL [newFL/ year]
2	4	206	0	1	0.1	0.3
7	17	1577	2	2	0	0
1	16	1415	1	1	0	0
8	25	1817	1	1	0	0
24	122	782	0	4	1.5	7.5
8	27	586	0	4	0.6	2
192	192	7220	3	3	0	0
221	648	17272	5	9	1.2	4.3
		16040	1	1		
29	29	1458	3	3	0	0
9	22	643	0	3	0.5	1.6
57	57	1997	4	3	–1.9	–1.9
4	18	338	0	2	0.3	1.3
654	822	43755	3	4	0.2	0.4
603	2388	27979	2	14	3.6	14.9
230	594	7901	0	14	4.2	6.3
351	396	11024	2	4	0.9	0.9
397	397	7231	1	1	0	0
		53948	2	2		
1876	2894	37752	4	14	6	7.8
311	311	7476	3	3	0	0
486	486	10917	6	6	0	0
925	1438	7605	0	3	1.6	3.2
705	705	6461	1	9	16	16
1492	1492	13914	12	16	7.7	7.7
211	211	2194	1	3	2.6	2.6
52	52	1414	2	2	0	0
1572	1572	20283	16	16	0	0
		33760	1	1		

Supplementary Figure 1: Overview over biomarkers during the complete follow-up and clinical outcome for all patients showing focal lesions. On the left side, a timeline for each patient displays all performed wb-MRIs. Symbols above the corresponding MRI indicate which biomarker was positive at which MRI. Additionally, the event terminating the observation period is laid out. On the right side, the mean and maximum values for SOG and dev-nFL, the maximal TTV and the nFL at first and last MRI are given for each patient.

Supplementary Table 1: Correlation between initial TTV and baseline disease parameters

Parameter	N	rho	p-value
M-Protein	32	0.21	0.2571
Plasmacell count	40	0.02	0.9173
Lactate dehydrogenase	48	-0.02	0.8711
Creatinin	57	-0.08	0.5757
Calcium	55	-0.12	0.3773
Platelets	48	0.00	0.9993
Leukocytes	48	-0.00	0.9821
B2MG	38	0.23	0.1632
Hemoglobin	57	0.02	0.8662
Albumin	52	-0.11	0.4440
C-reactive protein	36	-0.13	0.4352
